# Effects of Various Types of Expandable Graphite and Blackcurrant Pomace on the Properties of Viscoelastic Polyurethane Foams

**DOI:** 10.3390/ma14071801

**Published:** 2021-04-06

**Authors:** Rafał Oliwa, Joanna Ryszkowska, Mariusz Oleksy, Monika Auguścik-Królikowska, Małgorzata Gzik, Joanna Bartoń, Grzegorz Budzik

**Affiliations:** 1 Department of Polymer Composites, Faculty of Chemistry, Rzeszow University of Technology, PL-35959 Rzeszow, Poland; molek@prz.edu.pl; 2 Department of Ceramics and Polymers, Faculty of Materials Science and Engineering, Warsaw University of Technology, PL-02507 Warsaw, Poland; joanna.ryszkowska@pw.edu.pl (J.R.); monika.auguscik.dokt@pw.edu.pl (M.A.-K.); Malgorzata.gzik96@gmail.com (M.G.); Joanna.barton93@gmail.com (J.B.); 3 Department of Mechanical Engineering, Faculty of Mechanical Engineering and Aeronautics, Rzeszow University of Technology, PL-35959 Rzeszow, Poland; gbudzik@prz.edu.pl

**Keywords:** polyurethanes, graphite, natural filler, flame resistant, thermal stability, mechanical properties, chemical analysis

## Abstract

We investigated the effect of the type and amount of expandable graphite (EG) and blackcurrant pomace (BCP) on the flammability, thermal stability, mechanical properties, physical, and chemical structure of viscoelastic polyurethane foams (VEF). For this purpose, the polyurethane foams containing EG, BCP, and EG with BCP were obtained. The content of EG varied in the range of 3–15 per hundred polyols (php), while the BCP content was 30 php. Based on the obtained results, it was found that the additional introduction of BCPs into EG-containing composites allows for an additive effect in improving the functional properties of viscoelastic polyurethane foams. As a result, the composite containing 30 php of BCP and 15 php of EG with the largest particle size and expanded volume shows the largest change in the studied parameters (hardness (H) = 2.65 kPa (+16.2%), limiting oxygen index (LOI) = 26% (+44.4%), and peak heat release rate (pHRR) = 15.5 kW/m^2^ (−87.4%)). In addition, this composite was characterized by the highest char yield (m_600_ = 17.9% (+44.1%)). In turn, the change in mechanical properties is related to a change in the physical and chemical structure of the foams as indicated by scanning electron microscopy (SEM) and Fourier transform infrared spectroscopy (FTIR) analysis.

## 1. Introduction

Due to the possibility of modifying the properties of polyurethane foams (PUFs), they have been used in many industries for many years, including construction, automotive, furniture, etc. [1]. In most of these applications, high resistance to fire is required from the foams. However, the developed porous structure of open-cell flexible foams and the low density of the foams make them highly flammable. In addition, the chemical nature of polyurethane foams causes them to emit toxic and corrosive gases during a fire [2]. Therefore, it is important to provide PUFs with adequate fire resistance to reduce their risk to human life and property safety [3]. European Union regulations recommend the use of halogen-free compounds for flame retardancy [4]. These materials are subject to various requirements: limiting flammability, reducing the amount of smoke released, and no effect on the toxicity of gaseous combustion products, or its reduction; moreover, their introduction should not significantly affect the functional properties of the foams [1,4]. In the technology of polyurethane foams, reactive flame retardants are known—chemically bound to the polymer chain, among which a number of polyols containing phosphorus—in the main [5] or secondary chain [6], and nitrogen [7] can be distinguished. However, a more economical method of improving flame resistance is to incorporate additive flame retardants into the polyurethane matrix. Again, a very large group of additive flame retardants are compounds containing phosphonate [8,9], phosphinate/phosphite [10,11], phosphate [12,13], polyphosphate [14,15,16], phosphorus oxide [17], and 9,10-dihydro-9-oxa-10-phosphaphenanthrene-10-oxide (DOPO) derivatives [18,19], as well as boron [20,21] and hydroxides [22,23]. In addition, the mentioned flame retardants are often used together with nanofillers. Nanocomposites are a broad and continuously developing group of materials, including polyurethane foam technology [24]. However, polyurethane foams containing only nanofillers show a slight improvement in flame resistance [25,26]. As a result, nanoparticles are used with other commercial flame retardants directly added to the polyol [25,27] or used for the synthesis of new systems [28,29,30]. To obtain flame retardant polyurethane foams, expanding graphite is also used and well known as an independent, effective flame retardant [31,32,33]. During the combustion of foams with graphite, the scale increases due to the expansion of graphite, which creates a thermal barrier limiting oxygen diffusion and heat and mass transfer. The literature’s data indicate that the greater the expansion volume, the higher the probability of forming a more intumescent char [34]. Moreover, the graphite available on the market differs in particle size which also affects its effectiveness [35]. Li et al. [36] showed the addition of 30 parts per hundred of polyol by weight of graphite with an average particle size of 960, 340, and 70 µm, resulting in semirigid polyurethane foams with flammability class V0, V1, and HB75. A better flame-retardant effect for foams with larger grain size graphite results from the formation of a greater amount of char residue [32,37]. Moreover, the addition of approximately 30 parts per 100 of polyols by weight ensures that the oxygen index of rigid polyurethane foams is 25% [38,39]. However, the addition of graphite also increases the viscosity of the reaction mixture which may result in the deterioration of mechanical properties [32,33,37]. Therefore, in recent years, researchers have focused on finding synergistic effect of expandable graphite (EG) with other additive flame retardants [40,41,42] or nanofillers [43,44,45]. The main mechanism to improve the flame resistance is to increase the char yield [46,47,48].

Another important aspect related to PUFs industries is limiting their negative impact on the natural environment by transforming waste into resources in accordance with the circular economy guidelines [1]. One of the methods in line with the economic and legal requirements related to environmental protection is limiting the use of petrochemical raw materials in the synthesis of polyurethane foams by using polyols of natural origin obtained from plants, such as castor oil [49,50], tung oil [51,52], rapeseed oil [53,54], soybean oil [55], limonene [56], and orange peel [57]. Another method of reducing the negative impact of polyurethane foams on the natural environment is the addition of natural fillers, which improve the biodegradability and recyclability of the foams. These are different types of fillers and nanofillers, such as cellulose and lignocellulosic fibers [58], sisal fibers [59], eggshell wastes [60], date palm particles [61], walnut and hazelnut shells [62], esparto wool [63], and rice husk [64,65]. In additional to the benefits of waste reuse, plant-based fillers are also characterized by their low cost, low density, and ability to be easily processed and to be noncorrosive. These fillers can be used to improve the structural, mechanical, thermal, and acoustic properties of polyurethane foams [64,66,67]. However, in contrast to the advantages, the use of natural fillers brings some limitations related to increased flammability of foams, water absorption, or even deterioration of some mechanical properties [59,68]. One of the options is to use hybrid systems with other additives [63,69,70]. For example, Yun et al. [71] used cellulose-based flame retardants and expanded graphite to obtain hybrid polyurethane foams. The authors showed improved compressive strength of the hybrid foams, but the limiting oxygen index (LOI) and residue yield were lower than for foams containing only EG.

The growing number of publications on the methods of modification of polyurethane foams in terms of improving their flame resistance and the use of raw materials of natural origin in their synthesis indicate that these are important aspects of the development of polyurethane foam technology in the context of increasing their application possibilities. However, the cited publications mostly concern rigid polyurethane foams. Furthermore, some of the results of the cited works indicate that the use of natural fillers has an adverse effect on the properties of the foams that reduce their suitability. Therefore, the present work focuses on obtaining hybrid polyurethane foam composites that contain another filler or nanofiller, in addition to the natural filler. However, there is still insufficient information on the simultaneous use of graphite and a natural filler in the flexible polyurethane foam technology. Our previous work on the positive effects of using biomass waste from the food industry, including blackcurrant pomace for filling rigid polyurethane foams, indicates that the use of this filler in flexible polyurethane foams can improve the mechanical and thermal properties of these materials. On the other hand, the simultaneous introduction of graphite and blackcurrant pomace into the matrix can create completely new materials with wide application possibilities, in line with the current trends in the development of polyurethane foams.

Hence, in the present work, the influence of simultaneous application of graphite of different grain sizes and, consequently, different degrees of expansion, and of blackcurrant on the functional properties of viscoelastic polyurethane foams (VEF) belonging to the group of flexible polyurethane foams was investigated. Tests were carried out in terms of flame resistance, thermal stability, and mechanical properties, as well as the foam structure, to verify the applicability of blackcurrant pomace as a new natural foam filler. On the basis of the obtained results, it was found that blackcurrant pomace improves the functional properties such as flame resistance and compression strength of VEF through additive effect with graphite.

## 2. Materials and Methods

### 2.1. Materials

The components used to prepare the viscoelastic polyurethane foams are presented in Table 1.

Water was used as a blowing agent. For the modification of the foams, the following materials were used: expanded graphite with different particle sizes was purchased from Sinograf, Toruń, Poland (Table 2.), and blackcurrant pomace—product of fruit processing from the company AGROPOL Sp. z o.o., Góra Kalwaria, Poland. The pomace was ground in an impact mill and then dried. Drying of the filler was carried out at the temperature of 70 °C for 18 h. The particle size of this filler is 13–1400 µm. The particles of this filler contained approximately 6% water.

### 2.2. Preparation of Composites of Viscoelastic Polyurethane Foams

The foams were produced in one-step method from component A and component B. Component A was prepared from the abovementioned raw materials, which contained polyols, catalysts, surfactants, and foaming agent. The flame-retardant additives and/or blackcurrant pomace (BCP) were added to component A. Component B is an isocyanate mixture. The viscoelastic foams were made with the isocyanate (INCO) index = 0.9. Details information on foam formulation is the know-how of FAMPUR company (FAMPUR Adam Przekurat, Bydgoszcz, Poland).

After the raw materials were weighed into a component A, they were mixed with a mechanical stirrer for approximately 20 s at a speed of 3000 rpm. Then, component A was combined with component B and mixed for 10 s at 3000 rpm. The reaction mixture was poured into an open rectangular polypropylene mold 200 × 200 × 200 mm. The foam growth time was determined during the reaction. After the gelling process, the foams were annealed at 70 °C for an hour, then seasoned for 3 days at room temperature. After seasoning, the materials were cut into rectangular 50 × 50 × 50 mm pieces required for the tests, slices 5 mm thick and 20 mm thick. Chemical composition and designations of the foams are summarized in Table 3.

### 2.3. Methods

Growth time was defined as the time from mixing components A and B until the reaction mixture reaches its maximum volume (height). This time was measured with a stopwatch.

The apparent density (d) of the foams was determined in accordance with PN-EN ISO 845:2010. The weight of the foams was determined with an accuracy of ±0.001 g, and the dimensions of the samples were measured with an accuracy of ±0.01 mm.

Observations of the macrostructure of the composites were carried out using scanning electron microscopy (Hitachi TM 3000 SEM, Tokyo, Japan). Before the observations, the samples were dusted with a layer of gold with palladium. The observations were made using a voltage of 5 keV.

The chemical structure was analyzed using absorption spectra obtained with a Nicolet 6700 Fourier transform infrared spectroscopy (FTIR) spectrophotometer (Thermo Electron Corporation, Waltham, MA, USA) with an ATR attachment (attenuated total reflection). Three samples from each part were scanned 64 times each in the wavenumber range 4000–400 cm^−1^. The analysis of the spectra obtained was carried out in the Omnic 8.2.0.387 software (Thermo Fisher Scientific Inc., Waltham, MA, USA).

Thermogravimetric analysis (TGA) was performed using a TGA Q500 (TA Instruments, New Castle, DE, USA). Samples weighing 10 ± 1 mg were tested under nitrogen atmosphere, heating them at a rate of 10 °C/min from room temperature to 600 °C. The results were analyzed using Universal Analysis 2000 ver. 4.7A (TA Instruments, New Castle, DE, USA). For each type of composite, three measurements were made.

Differential scanning calorimetry (DSC) was performed on a DSC Q1000 of TA instruments (New Castle, DE, USA). Measurements were made in a helium atmosphere in airtight aluminum crucibles. About 6 mg of samples were heated at a rate of 10 °C/min from −80 °C to 250 °C.

The compression test was carried out in accordance with the PN-EN ISO 3386-1:2000 standard on the Zwick Z005 testing machine (Zwick Roell Group, Ulm, Germany). Each sample was compressed four times by 75% height. The time between consecutive measurements was 5 min for the foam to relax the stresses and return to the dimensions before deformation. We read the values of compressive stresses during loading and unloading, thus obtaining a hysteresis loop. Based on the results from the fourth compression process, the foam comfort factor (Cf) was determined, i.e., the ratio: stress at 65% load/stress at 25% load and the stress when loading samples at 40% strain, which is defined as the foam hardness (H).

The permanent deformation under compression was determined on 50 × 50 × 50 mm samples, in the direction of foam growth, in accordance with the PN-EN ISO 1856:2018-09 standard. The measured samples were placed between two metal plates, compressing them to 50%, 70%, or 90% of the original height. The compressed samples were annealed for 22 h at 70 °C, and then they were taken out, and after 30 min at room temperature the height was again measured. The percentage reduction in the height of the samples was calculated, thus determining the permanent deformation.

The foams were also characterized using a mass loss calorimeter (MLC) from Fire Testing Technology Ltd. (East Grinstead, UK) in accordance with ISO 13927. The samples with the dimension of 100 × 100 × 10 mm were tested by applying a heat flux of 25 kW/m^2^ and the distance from the ignition source of 25 mm.

The limiting oxygen index (LOI) for the polyurethane foams was determined according to the standard EN ISO 4589 at room temperature using an instrument of FTT Ltd. (East Grinstead, UK). Ten specimens were measured for each viscoelastic polyurethane foams (VEF) type.

The flammability tests by using UL-94 method were carried out in a chamber produced by FTT Ltd. (East Grinstead, UK). The measurements were made according to the standard PN-EN 60695-11-10 with vertical and horizontal sample beam position and a methane fed burner of 25 mm height. Each type of test was performed on at least three samples from each series.

## 3. Results and Discussion

### 3.1. Physical Properties of Polyurethane Foams

**Figure 1:**
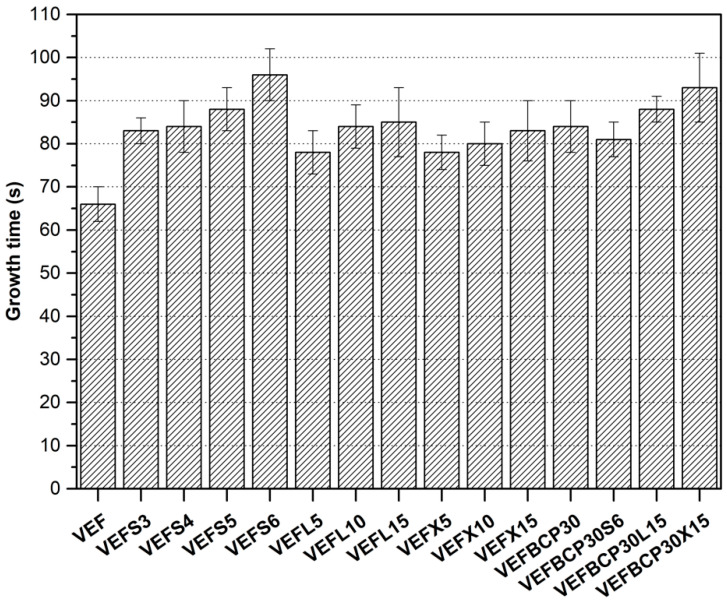
The growth time of VEF composites.

In Figure 1, the change in the growth time of foams with expandable graphite of different particle sizes is showed. The presented results show that the growth rate of the foams decreases after the introduction of graphite, which may be a result of the increase in the viscosity of the reaction mixtures after the introduction of graphite. The results presented in [72] confirm that decrease in the reactivity of polyurethane (PUR) systems with expandable graphite is associated with an increase in the viscosity of polyol graphite blends. Moreover, the reactivity of VEF system decreased with the particle size of graphite. In turn, the introduction of 30 php of BCP into the VEF increases the growth time by about 27%, and the total introduction of 30 php of BCP and 15 php of EG causes an increase in growth time of approximately 33% (EG L) and approximately 44% (EG X). Such a significant increase in growth time confirms previous conclusions regarding the increase in viscosity of these systems.

### 3.2. FT-IR Analysis

**Figure 2:**
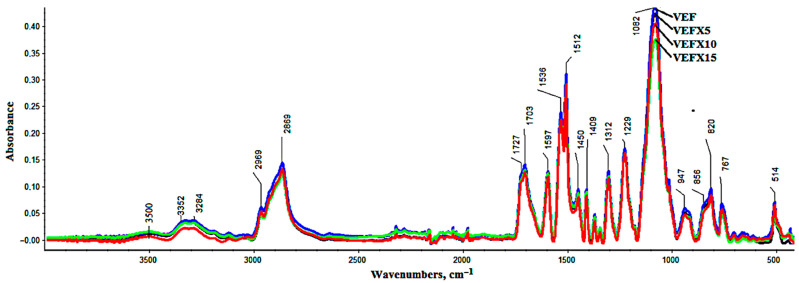
FT-IR spectra of unmodified foam (VEF) and modified with different content of EG 399: VEFX5, VEFX10, and VEFX15.

Figure 2 shows representative spectra for the obtained samples. The band near wave-number 3500 cm^−1^ is derived from the group –OH from water or unbound polyols. Wavenumber 3352 cm^−1^, 3284 cm^−1^ originates from the stretching vibration, symmetrical and asymmetrical, assigned to the N–H bond. Clearly, the outlining range of wave numbers 2869 cm^−1^ and 2969 cm^−1^ originates from stretching vibrations within group –CH2 in the soft segments formed from polyols [73]. In all analyzed samples, there were also observed bands derived from bond vibrations of C=O (1701, 1709, and 1724 cm^−1^), C=C from the aromatic ring (1598 cm^−1^) bending and deformation vibrations derived from N–H bond HNC=O (1536 and 1512 cm^−1^), CH_3_–C (1450 cm^−1^), –O–CH_2_ (1409 cm^−1^), and νasym CO/sym within the group –NCO–O (1229 cm^−1^) in –C–O–C– group [73]. In the range around 767 cm^−1^, the band represents a C-–H bond from the aromatic ring.

**Figure 3:**
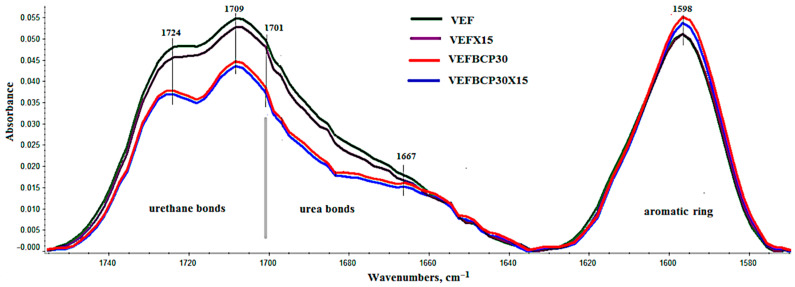
Region of C=O of FT-IR spectra of unmodified foams (VEF) and modified with 15 php of graphite 399 (VEFX15), with 30 php of blackcurrant pomace (VEFBCP30) and the mixture of graphite 399 and blackcurrant pomace (BCP) (VEFBCP30X15).

The introduction of graphite causes a distinct change in intensity and band shift. The introduction of graphite causes slight changes in the intensity of the bands in the FTIR spectrum. On the other hand, the introduction of BCP causes significant changes in the intensity of the bands coming from vibrations of the C=O groups in the urethane and urea bonds and the intensity of the bands coming from the vibrations of the aromatic groups (Figure 3).

The introduction of 30 php of BCP to PUR systems is associated with the introduction of an additional amount of OH groups on the surface of the filler but also an additional amount of water bound in the filler particles. Both factors influence the change in the isocyanate index (RNCO/OH) as they may influence affecting the consumption of NCO groups [74]. The introduction of BCP may change the ratio of urethane groups to urea groups, which is indicated by the change during the analyzed spectra in the range of 1635–1755 cm^−1^, and the results presented in the works of other authors [32,50]. Often, the introduction of fillers also causes changes during phase separation in PUR, and consequently, in the properties of PUR foams. The course of the phase separation process with the help of FTIR can be analyzed on the basis of the interpretation of the N–H region and C=O region (Figure 2). Since interpretation of the N–H region (3250–3400 cm^−1^) is rather difficult; thus, our study focused on C=O region (1655–1750 cm^−1^) (Figure 3).

**Figure 4:**
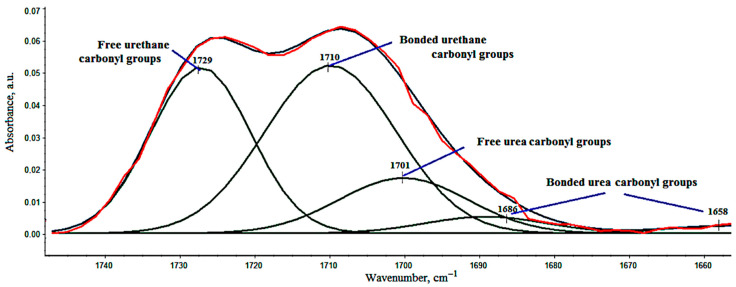
Region of C=O of FT-IR spectra of VEFL15 after decomposition in Gaussians.

Analysis of this region by decomposition in Gaussians results in five bands with maxima at ca. 1729, 1710, 1701, 1686, and 1658 cm^−1^ (Figure 4). Five different bands corresponding to free urethane (1729 cm^−1^), bonded urethane (1710 cm^−1^), free urea (1701 cm^−1^), monodentate urea (1686 cm^−1^), and bidentante urea (1658 cm^−1^) in disordered phase can be observed [75]. The vibrations at 1686 and 1658 cm^−1^ are assigned to hydrogen-bonded urea groups [76].

Since the introduction of fillers, i.e., EG, and BCP, may affect the formation of a network of hydrogen bonds between urethane or urea groups and carbonyls in PUR [77], based on the size of the surface area of each band, the share of hydrogen bonds connecting rigid segments in VEF (R) was calculated, including the degree of phase separation (DPS) and the share of urethane and urea bonds in foams (UR and UA, respectively). The calculation method is presented in the paper by Mazurek et al. [78], and the results of the FTIR analysis of selected foams are presented in Table 4.

The introduction of EG and BCF causes an increase in the number of hydrogen bonds connecting the rigid segments in the hard phase of the analyzed foams, which causes the degree of phase separation in VEF to increase. Presumably, EG particles increase the thermodynamic incompatibility favoring the growth of DPS, and BCP particles favor the formation of more bonds between rigid segments [1]. The larger the EG particle size, the smaller number of hydrogen bonds joining the rigid segments. Larger EG particles limit the mobility of VEF macromolecules, which limits the possibility of hydrogen bonding between the segments. The addition of BCP and EG with different particle sizes to VEF increases the proportion of hydrogen bonds connecting the rigid segments. It is likely that the introduction of EG into VEF with BCP increases the thermodynamic incompatibility of both additives with VEF matrix, which leads to increased phase separation. Similar changes in DPS were observed after mint was introduced into VEF [79].

EG is a typical graphite intercalation compound with a flake-like graphite structure intercalated by sulfuric acid or nitric acid between its graphite layers [32,36]. During storage, EG can absorb H_2_O, which is favored by its structure. The EG used in the process of synthesis of the foams was not dried, which resulted in the increase of urea bonds in VEF with EG. An increase in the share of urea bonds was also observed after the use of BCP and BCP and EG mixtures. BCP particles easily absorb water and contain the so-called bound water, which causes that the share of urea bonds in the formed VEF matrix increases. The rate of reaction of NCO groups with H_2_O leading to the formation of urea bonds is greater than the rate of reaction of NCO groups with OH groups of polyols, which leads to the formation of urethane bonds; therefore, the share of urea bonds in VEF increases. The addition of additional water to the PUR system from EG and BCP causes the actual INCO of the system to be lower than that assumed during the calculations. This change in the chemical structure of the foams affected the changes in their physical structure (SEM analysis) as discussed in Section 3.5.

### 3.3. Thermogravimetric Analysis

**Figure 5:**
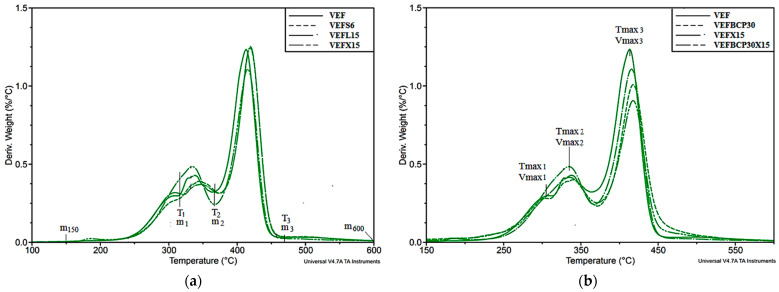
Mass change derivative curves (DTG) curves of VEF foams containing: (**a**) EG with different particle sizes, (**b**) BCP, EG X, and a mixture of BCP and EG X.

Based on thermogravimetric analysis in a nitrogen atmosphere, the influence of the addition of graphite and BCP on the thermal stability of VEF composites was determined. Changes in material properties were analyzed on the basis of the thermogravimetric curves (TGA) and mass change derivative curves (DTG) presented in Figure S1 and Figure 5, respectively, and data were summarized in Table 5. Based on the curves, the initial decomposition temperature (T_5%_), which is the temperature at 5% weight loss, the subsequent degradation stages Δm_i_, the temperature of the maximum degradation rate of each of them (T_maxi_), and the maximum degradation rate of these stages (V_maxi_) were determined.

In accordance with the literature [80], the thermal degradation procedure for VEF foams includes three stages in nitrogen (Figure S1). It was observed that up to the temperature of 150 °C (m_150_), there is a slight 0.3% change in the weight of the unmodified polyurethane foam, and then its thermal degradation begins; the course of which indicates the separation of the soft and hard polyurethane phases. In flexible polyurethane foams, agglomerates are formed consisting of rigid segments formed by urethane and urea bonds [81,82]. Urea domain agglomerates of urethane domains remain scattered or form clusters in the form of ovals [83]. The first stage of unfilled foam degradation runs in the range of 150–315 °C corresponding to a DTG peak at 304 °C (T_max1_) and the weight loss of 14.1% (Δm_1_). At this stage, decomposition of the residual polyol component and isocyanate unbound in PUR macromolecules, and the decomposition of the hard PUR phase composed of urea bonds forming spherical agglomerates. The second stage of degradation in the temperature range 315–370 °C corresponding to the T_max2_ and Δm_2_ of 338 °C and 18.6%, respectively, is related to the degradation of urethane bonds. The third stage (370–450 °C) with T_max3_ = 413 and Δm_3_ = 57% is attributed to the pyrolysis of the residual polyether chain [84,85]. Moreover, the maximum rate of degradation increased in the subsequent steps from 0.30%/°C in the first step to 0.43%/°C in the second and 1.23%/°C in the third step, which is attributed to the higher content of soft segments [80]. In turn, the carbon residue at 600 °C amounts to 10%.

**Figure 6:**
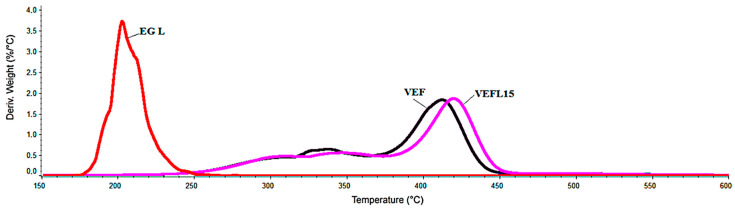
Comparison of DTG curves: EG L, VEF, and VEFL15.

In the case of some composites, it was also observed that the process of thermal degradation occurs in three stages, as there is also a separation of the hard phase, the phase rich in urea bonds (stage 1), and urethane (stage 2). The VEFL15 and VEFX15 composites are an exception, because the DTG curves do not show any inflection at the border between the first and second degradation stages. Therefore, for this type of foam, the mass change—Δm_1_ (end of the first stage of degradation) was determined for the same temperature for which this change was determined in foams with a clear boundary between stages 1 and 2. The effects related to the decomposition of EG may overlap with the effects of decomposition of the rigid segment. To explain this assumption, TGA analysis of EG L graphite was performed. As it was shown during this analysis, this graphite decomposes in the range of 168–260 °C, with the maximum degradation rate at 202 °C of 3.7%/°C, and after it, degradation at 600 °C remains 6.8% by weight. Thus, the graphite degrades before the decomposition of the rigid segments begins (Figure 6).

The data summarized in Table 5 show that at 150 °C the weight loss of the EG foams is the same as that of the unmodified foam. Contrary to the literature’s data [18,20,21,32], the temperature at which there is a 5% weight loss of foams containing only graphite is higher compared to unmodified foam, regardless of the size of the graphite introduced. It proves that heat and oxygen exchange is inhibited by the graphite structure, which covers the polymer matrix, improving its thermal stability [16]. As a result, the temperature of the maximum degradation rate in the first stage is shifted toward higher values compared to the reference sample and is in the range of 305–311 °C. On the other hand, the loss of mass in the first stage of degradation in most of the foams with graphite was lower compared to the VEF foam. This is due to the fact that the graphite flakes that cover the polymer layers expand upon heating and probably limit the growth of volatile products formed during the initial phases of matrix decomposition (rigid segments and oligomeric MDI fragments) and thus improve the thermal stability of the residue, as confirmed by T_max1_. Among these samples, the smallest Δm_1_ were found for foams containing 5 and 10 php of graphite L, whose T_max1_ was the highest and amounted to 310 and 311 °C, respectively. Moreover, a relationship was observed between the type and amount of graphite and the weight loss in the first stage of pyrolysis, because with the increase in the graphite L and X content, the weight loss increased; while for graphite S, this relationship was reversed. In the second stage of decomposition, the foam containing 5 php of L graphite was also characterized by the highest value of T_max2_ = 348 °C, and the change obtained was even more pronounced compared to the unmodified foam. Despite this fact, the percentage weight loss of this foam was the greatest in relation to VEF as well as other foams with graphite, which were also characterized by higher values Δm_2_ and T_max2_ compared to VEF. The weight loss in the subsequent stages of composites decomposition is related to their composition presented in Table 2. The introduction of EG flame retardant, BCP filler, or their mixtures increases the total weight loss in the first and second degradation stages, which indicates that more urea bonds are formed in these materials and urethane. This change is due to the introduction of more water into the substrate mix. In the last stage of pyrolysis, in relation to the VEF sample, composites containing graphite were again characterized by an increased value of T_max_ and Δm_3_. At this stage, the smallest increase in T_max_ (415 instead of 413 °C) but at the same time the smallest weight loss Δm_3_ (49% instead of 57%) was observed in the case of foam containing 15 php graphite X. As a result, the residual after pyrolysis was higher by 58% compared to the VEF, reaching 15.8%. For the remaining foams with graphite, an increase in carbon residue after pyrolysis was also noted. Moreover, a relationship was observed between the amount and type of added graphite and the value of carbon residue after pyrolysis, as for the S, L, and X graphite foams it was in the range of 11.2–11.9, 11.7–12.8, and 11.4–15.8%, respectively. On the basis of these values, it was found that the addition of graphite promotes the formation of a carbon layer, the efficiency of which is greater for graphite with a larger particle size. Moreover, EG X graphite has a greater expansion coefficient than EG L, which may be a decisive factor influencing the course of the degradation process. Certain relationships were also observed between the rate of degradation and the content and the type of graphite, especially in the second and third stages, because by increasing its content, a tendency to increase and decrease the degradation rate, respectively, is observed. In the first stage, it was lower than for the unmodified foam and ranged from 0.25–0.30%/°C, while in the second stage the degradation rate was 0.37–0.48%/°C, and 1.11–1.26%/°C in the third stage.

The significant reduction in the rate of decomposition in the third stage of foams containing 15 php of graphite indicates that this amount produces an expanded carbon layer of EG, which is a physical barrier limiting the decomposition of viscoelastic polyurethane foam [21]. This is also confirmed by the flammability results, which were the most favorable for foams containing 15 php of L or X graphite and in particular for the VEFX15 foam. In the case of semirigid foams with the addition of expanded graphite (the research results of which are presented in the work of Li et al. [32] as in the tested foams) the occurrence of three stages of degradation was observed but occurred in place at lower Vmax: the first 0.16–0.22%/°C, the second 0.20–0.28%/°C, and the third 0.85–1.05%/°C.

In the case of foams containing only BCP, the thermal degradation process also consists of three stages, while the additional addition of graphite changed the degradation course to two peaks in the DTG thermogram. The introduction of BCP filler increases m_150_ in relation to other foams (m_150_ = 1.4–1.7%), which is the result of water loss from BCP (m_150_ = 5.8 wt. %). The thermal stability of foams containing BCP and BCP with graphite significantly decreased compared to the unmodified foam. The temperature at which the mass loss of the sample reached 5 wt. % of the VEFBCP30 was lower by 26 °C, compared to the unmodified foam, which is related to the organic nature of the filler, whose initial decomposition temperature T_5%_ was 127 °C. The decrease in thermal resistance may also be related to the more porous structure with a differentiated structure of cells visible in the SEM photos (Figure 9a) [86]. Despite this, the first stage of decomposition was characterized by a DTG peak and a weight loss at the level of unfilled foam, as the difference was only 2 °C and 0.2%. Moreover, the temperature of the maximum degradation rate in the second and third stages is greater than that of VEF foams, which may indicate that BCP promotes the formation of a thermally stable coke that inhibits heat and mass flow. This is also evidenced by the residue after thermolysis of BCP itself, which was 24.5%. This is also confirmed by the recorded significantly lower weight loss and the maximum rate of weight loss of the VEFBCP30 foam, by 11.8 wt. % and 0.22%/°C, respectively, compared to the reference sample. As a result, the addition of BCP improves the char yield (12.7% instead of 10%), which is one of the mechanisms to improve the flame resistance of these foams. It is worth noting, because the addition of natural fillers often reduces the thermolysis residues [87,88]. Similar conclusions can be drawn when analyzing thermograms of foams containing both BCP and graphite. Among the foams with hybrid systems, the highest values of T_5%_, T_max2_, and T_max3_ were recorded for the VEFBCP30S6 sample, which coincides with the results for the foam containing only 6 php of graphite S. The addition of 6 php of graphite S next to BCP also caused a further decrease in the degradation rate, reaching in the third step 0.97%/°C. In contrast, the decomposition residue at 600 °C was 14.2%, an increase of 42 and 11.5%, for VEF and VEFBCP30, respectively. On the other hand, the VEFBCP30L15 and VEFBCP30X15 foams showed a greater increase in scale efficiency, reaching 15.6 and 17.9% of the carbon residue, respectively. The foam containing 30 php BCP and 15 php graphite X also had the lowest decomposition rate in step 3, V_max3_ = 0.91%/°C, which was a change of 26, 10, and 18%, compared to VEF, VEFBCP30, and VEFX15, respectively.

Summarizing, as shown in Figure 5a,b, the introduction of EG and BCP causes changes during the degradation stages related to the decomposition of urethane and urea bonds, which may also result from the course of phase separation in these materials. These changes may result from changes in crosslinking density and may affect the morphology of foams, as well as the thermal and mechanical properties. Moreover, the TGA analysis of foams containing BCP and graphite shows, first of all, a reduction in the degradation rate and an improvement in scale efficiency in relation to VEFBCP30 and foams containing graphite, which proves the additive effect of these additives in the creation of a thermally stable solid residue. Such a scale is better limiting the heat and mass flow from the polymer surface, which significantly affects the behavior of these foams under fire.

### 3.4. Thermophysical Properties

**Figure 7:**
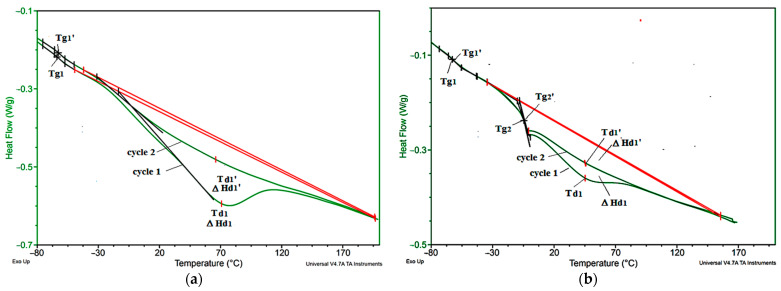
DSC thermogram of selected sample: (**a**) VEF and (**b**) VEFX15.

Characteristic temperatures of the tested materials were analyzed with the use of DSC. Sample DSC thermogram curves are shown in Figure 7.

On the basis of the DSC thermogram, it was observed that in the VEF foam during the first heating, there is an inflection typical of the glass-transition temperature (T_g1_) in the soft phase of polyurethane and an endothermic peak resulting from the order disorder in the hard phase PUR occurring at the minimum temperature (T_d1_) associated with a change in enthalpy (ΔH_d1_). During the second heating cycle, similar glass-transition phenomena (T_g1′_) and order disorder transformation (T_d1′_, ΔH_d1_) are observed. Similar transformations were observed in the BCP foams and in the foams with the BCP and EG mixture. On the other hand, in foams with graphite with EG 296 and EG 399, there is an additional change related to the glass transition in the soft phase, in which the scattered hard domains (T_g2_) remained. This transformation is also visible during the second heating of the tested foams (T_g2′_). On this basis, it can be concluded that the introduction of large EG particles changes the course of the phase separation process in foams. The values of the parameters determined from the DSC thermograms in the first and second foam heating cycle are summarized in the Table 6.

The glass-transition temperature of the soft phase of the tested foams (T_g1_) ranges from −59.6 to −64.2 °C, in the second heating cycle, and it slightly changes by no more than 2 °C. In the case of EG foams with a large particle size, the glass-transition temperature of the phase with more dispersed hard domains (T_g2_) is −3–−6 °C and does not change in the second heating cycle.

In the case of the analyzed EG foams with large particle sizes (EG L, and EG X), there is a tendency to decrease the mobility of flexible segments after introducing more particles, which is indicated by a change in T_g1_ and a change in T_g2_. For this group of foams, along with the introduction of a larger amount of EG additive, an increase in the enthalpy of the change order–disorder determined in the first and second heating cycle is also observed.

In the case of BCP foam, the T_g1_ is clearly decreased and the transformation temperature order–disorder increases. This indicates that during the phase separation process, a greater amount of ordered hard phase bound by hydrogen bonds was formed in these materials, the dissociation of which requires more energy. Such a change may be the result of the formation of a greater number of urea bonds formed in these materials as a result of introducing more water in BCP, which is confirmed by the results of the FTIR analysis presented in Table 4.

### 3.5. SEM Analysis of Brittle Fracture Surface of VEF Composites

**Figure 8:**
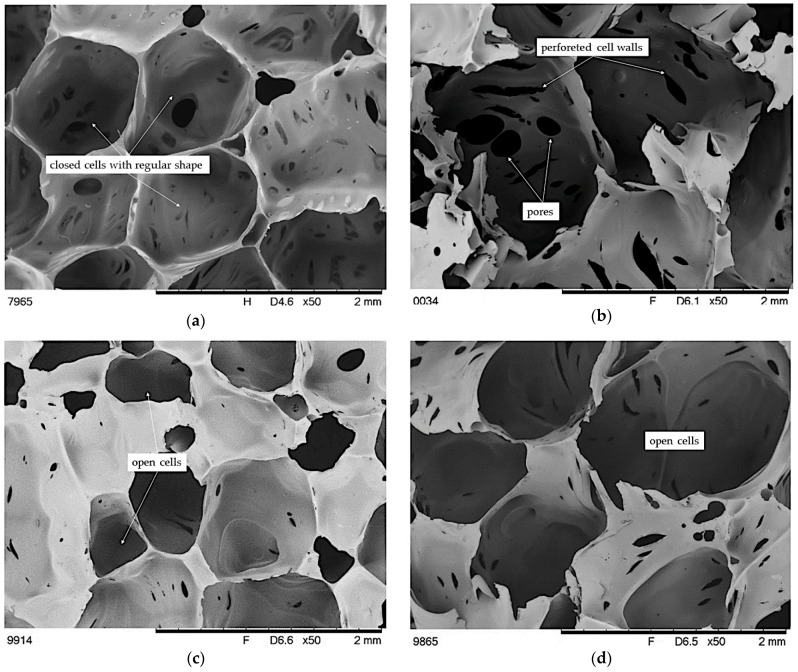
SEM microphotographs of brittle fracture of foams: (**a**) VEF, (**b**) VEFS5, (**c**) VEFL15, and (**d**) VEFX15.

The consequence of differences in the morphology of the polyurethane matrix of the composites, the type, and amount of EG used are differences in the pore structure of the foams (Figure 8). In turn, the pore structure of the foams significantly influences the mechanical and physical properties of the foam. Analysis of SEM photos of brittle fractures of polyurethane foams shows a typical morphology for foams, as the cells of unmodified foam are approximately symmetrical polyhedral of regular shape [21]. Furthermore, most cells are close, and no collapse in the cell morphology was observed. On the other hand, the addition of graphite causes an increase in the mean cell size. Moreover, the addition of EG caused an increase in the pore size distribution of the foams and an increase in the number of holes connecting the pores. It can also be observed that the structure has become more porous, and the cells are more open and perforated. Similar conclusions were reported by [37]. According to [36], this is because EG releases some gases during the highly exothermic reaction between polyols and PMDI and the –OH surface of the group of EG. Interestingly, these changes are especially noticeable in the case of the foam containing graphite with the smallest grain size. This is contrary to the literature data. According to Gao et al. [39], who used graphite with an average particle size of 180 µm, the dispersion of additives with a small particle size does not significantly affect the size and homogeneous integrity of foams, because most of the particles are in the cell wall or backbone of the polymeric matrix. On the other hand, the addition of graphite with an average particle size has the smallest effect on the size and homogeneous integrity of PUR foams.

**Figure 9:**
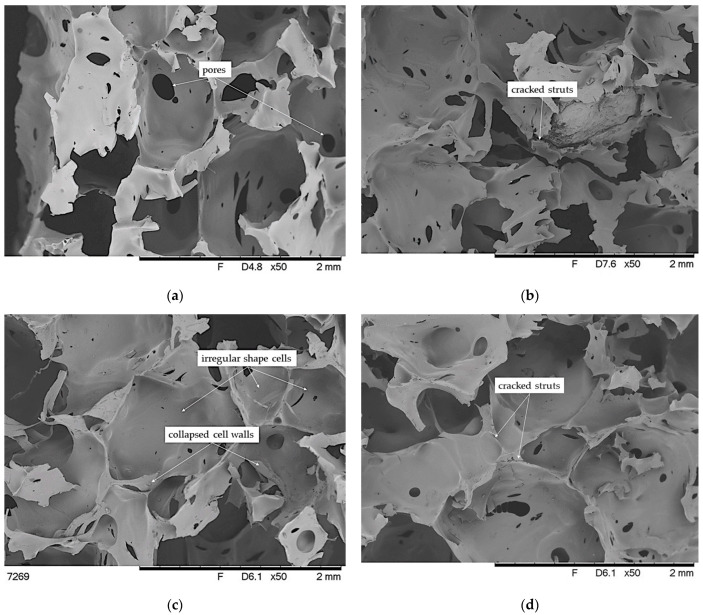
SEM microphotographs of brittle fracture of foams: (**a**) VEFBCP30, (**b**) VEFBCP30S5, (**c**) VEFBCP30L15, and (**d**) VEFBCP30X15.

The introduction of BCP and mixture of BCP with EG causes a marked change in the pore structure of the foams compared to unfilled foam and foams containing only graphite. The high content of BCP causes the pores to have irregular shapes and size, assuming the shape of polygons (Figure 9). In addition, cracks of some struts and collapsed cell walls are visible. This is due to the large size of BCP particles and their content, as they can act as a cell-opening agent and disrupt the foaming process, which in turn affects the formation of a diverse and weak cell structure [89]. This can be explained by the fact that, along with BCP, significant amounts of –OH groups present on the surface and inside the filler are introduced (FTIR analysis), which disturbs the reaction between functional groups and causes the formation of spots in the mixture with differentiated growth of foam cells. In turn, the additional introduction of graphite of various sizes intensifies this effect. The structure of the foams is more diverse with the advantage of smaller pores compared to foams containing only graphite. As it has been described [87,88], the fillers can act as nucleation sites for cell growth, which, combined with the increase in the viscosity of the reaction mixture, causes the appearance of local internal stress or unbalanced foam growth and the creation of a collision and collapse in the target system.

### 3.6. Mechanical Properties of VEF Composites

Table 7 summarizes the results of the apparent density analysis of foams and the mechanical properties of foams determined in the compression test. The results are the arithmetic means of five tests for each type of foam. The introduction of EG causes the apparent density of the foams to increase. The greater the amount of EG introduced, the greater the density of the foam. The density also increases after the introduction of BCP and its mixture with EG. The increase in foam density results in a significant density of fillers: the density of expandable graphite is approximately 2200 kg/m^3^, with an apparent density of approximately 990 kg/m^3^ of BCP [90]. On the other hand, the composites containing EG and BCP have a much lower apparent density than would appear from the filler densities, which indicates that these materials contain more pores than the reference sample and confirms the SEM analysis which showed the formation of larger pores for the filler containing composites.

Based on the foam compression test, the comfort factor and hardness were determined, which is defined as the compressive strength at 40% foam deformation. The obtained results show that the addition of graphite increases the hardness of the foams in relation to the reference sample. The exception is foams containing 5 php of graphite, regardless of its grain size. This may be due to the uneven graphite content in these samples in the entire sample volume. On the other hand, regardless of the type of EG, the hardness of the foam increases with the increase of its content, which is the opposite tendency to the corresponding literature data on rigid polyurethane foams [32,34,37]. It can be concluded that the increase in compressive stress at 40% strain results from the increase in the stiffness of the flexible polymer matrix and the limitation of the mobility of macromolecules after adding larger amounts of graphite. On the other hand, the cell morphology had a smaller impact on the obtained results, as the SEM analysis showed an increase in the number of holes in the cell walls of the graphite-containing foams, which act as points of stress concentration and initiation of matrix fracture. Moreover, the obtained results do not indicate any correlation between the foam density and their hardness, which confirms the above thesis of strengthening viscoelastic polyurethane foams.

In contrast, the BCP foam exhibits compressive stress at 40% strain at the reference sample level. On the other hand, the additional introduction of 6 php of graphite with grain size S causes a decrease in the value of this parameter by 8% compared to the unmodified foam, which is the lowest value among the produced foams. However, the containing 15 php of L and X graphite foams have the highest hardness of 2.51 and 2.65 kPa, respectively, which is a change of 10 and 16% compared to the reference sample. Such a change may also be related to an increase in the stiffness of the matrix caused by the increase in crosslink density resulting from the presence of additional hydroxyl groups introduced with graphite and BCP. On the basis of the obtained results, a slight increase in the comfort factor of the foams after the introduction of graphite was found, and there is a tendency to increase the comfort factor along with the increase in their quantity. Moreover, a comparison of the results of the comfort factor of foams with the addition of the largest amount of individual types of graphite indicates slightly higher values for the foams with the largest graphite size and slightly lower for the foams containing the smallest size graphite. For foams additionally containing a natural filler, a significant increase in this parameter was noted. In these systems, a clear influence of BCP on the value of the comfort factor was observed, because regardless of the addition and the type of graphite, it was in the range of 2.60–2.66, i.e., in the range described as the most desirable (2–3). For many foam applications, the deformation of the foam after compression is essential. The smaller size of the EG particles, the more favorable their effect on the compression set deformation of the foam. Interestingly, advantageously low permanent deformations were observed for the VEFBCP30X15 foam, which was also characterized by the highest hardness, which can be explained by the obtained morphology with large cells capable of reversible deformation in a wide range.

### 3.7. Flammability of VEF Composites

#### 3.7.1. Limiting Oxygen Index and UL94

To test the behavior of the foams under the influence of fire, the oxygen index was determined, which is a basic and important parameter for assessing the flame resistance of polymer materials. We decided to describe the results for foams containing the highest amounts of graphite with a particular grain size, as such amounts were added together with the BCP. Based on the results summarized in Table 8, it was found that the addition of graphite causes an increase in the oxygen index of viscoelastic foams. Moreover, it was observed that the obtained LOI value was significantly influenced by the size of the graphite used, because the highest value of the oxygen index, 23.6% O_2_, was found in the foam containing 15 php of graphite with the largest particle size (399 µm). In turn, the foam containing 15 php of graphite with an average particle size of 290 µm reached the oxygen index at the level of 22%, which was a change of 3% O_2_ in relation to the unmodified foam. Similar conclusions were described in other works [32,37]. According to the literature [34,36], the difference in the oxygen index values could also be influenced by the expansion coefficient of individual graphite. It is particularly important because due to the flake structure of expanded graphite, it has the features of a typical intumescent flame retardant. In addition, sulfuric acid, nitric acid, phosphoric acid, and other acidic substances are usually introduced into the spaces between the layers, which lead to the formation of CO_2_, SO_2_, H_2_O, and other gases when the foams are burned. These gases dilute the gaseous products of combustion of the polymer, thereby lowering the temperature of the polymer surface. In addition, the gases released cause the EG flakes, formed during the thermal expansion of EG, to form a scale layer on the polymer surface, as confirmed by TGA analysis. This layer formed on the surface of the foam constitutes a physical barrier to the flow of energy between the burning layer and the rest of the polymer, thus delaying its further burning. Such a mechanism of action of EG in the case of the tested foams is also indicated by the reduction of the degradation rate in the third stage of foam decomposition (Table 5). As a result, it is assumed that graphite with a smaller particle size, especially about 96 µm, was not able to produce a sufficient barrier layer, which translated into a slight increase in the oxygen index of VEFS4 foam in relation to VEF. Additionally, this effect may be magnified by the difference in foam structure (SEM analysis of brittle fractures).

On the other hand, the addition of 30 php of BCP to the foam resulted in a slight decrease in the oxygen index from 18 to 17.6%, despite the fact that the TGA analysis showed the ability to form a thermally stable carbon. Therefore, the decrease in the oxygen index may be related to the more porous structure of the foams (SEM analysis), which makes it much easier for the flame to migrate deep into the material even though the foam density has increased significantly. However, compared to the literature data, the observed change was small, especially since we added as much as 30 php of BCP [86]. To solve this problem, additionally 15 php of L and X graphite was introduced, which resulted in a significant increase in the oxygen index to 23.7% and 26%, respectively. The obtained values confirm the synergistic effect of these additives in terms of increasing the oxygen index value, as well as the strong relationship between the scale efficiency and flame resistance. This synergistic effect can be mainly attributed to the promotion of the formation of a stable char with high yield by BCP and graphite (TGA analysis), because as indicated by SEM microphotographs, the addition of BCP, and graphite simultaneously enhanced the effect of forming a more differentiated structure with numerous holes and cracks, which is more susceptible to the flame.

The results obtained during the horizontal smoking test correspond to the results of the oxygen index. The unmodified foam burned completely at an average rate of 43.5 mm/min. Interestingly, the sample containing graphite S burned at a higher rate, reaching 70 mm/min. This confirms that graphite with a small particle size does not form a sufficient barrier layer, which may additionally be hampered by the significantly porous structure of this foam in relation to other foams with graphite. On the other hand, the remaining foam containing graphite with a larger grain size did not burn completely, as the flame was extinguished within 10 s after crossing the first marked line.

As would be expected, the foam containing 30 php of BCP burned more rapidly compared to the unmodified one but within the speed range classifying it as HB75. On the other hand, the addition of graphite L caused the foam to burn only 15 s after crossing the first measuring line, which gave the HB40b flammability class. In turn, the flame of the VEFBCP30X15 foam was stopped before the first measuring line, which once again confirmed the best flame-retardant effect of graphite with the largest particle size and expansion coefficient together with BCP. A similar direction of change after the application of EG was observed in the foams described in [91].

#### 3.7.2. MLC Analysis

**Figure 10:**
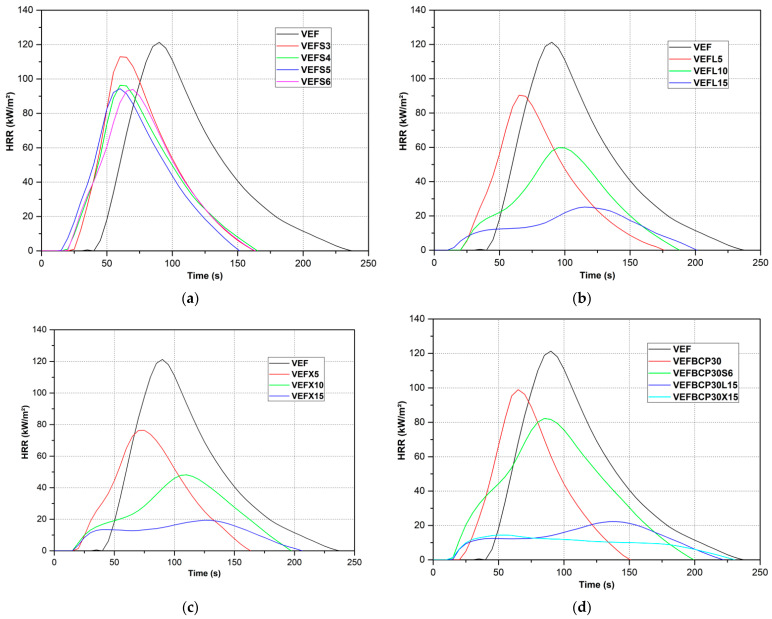
Heat release rate (HRR) curves as a function of time during flammability tests performed in the MLC calorimeter of unmodified polyurethane foam and foam composites containing: (**a**) EG S, (**b**) EG L, (**c**) EG X, (**d**) BCP, and BCP with EG S, EG L, and EG X.

To analyze in more detail the influence of the proposed additives on the flammability of the foams, flammability tests were carried out using the cone microcalorimeter MLC equipped with a thermopile. On the basis of the study, the basic parameters determining the behavior of the foams during burning were directly determined, such as heat release rate (HRR); peak heat release rate (pHRR); total heat released (THR); time to ignition (TTI); percent mass loss (PML); mass loss rate (MLR); and effective heat of combustion (EHC), which are summarized in Table 9. The results are the arithmetic means of three tests for each type of foam. Additionally, Figure 10a–d shows a representative curve for each replicate test.

Based on the recorded HRR curves as a function of time, it was found that the addition of graphite reduced the ignition time of the foams (Figure 10a–d). The unmodified foam ignited 36 s after the start of irradiation, while the ignition time of the graphite containing samples ranged from 6–18 s, depending on the amount and type of graphite. The recorded ignition time of the unmodified foam is much greater than that obtained by other scientists [20,34], which is related to the visible collapse of the viscoelastic foam structure before ignition due to its melting. As a result, the distance between the cone and the surface of the sample decreased, which extended the ignition time. In turn, the reduction in the ignition time of foams containing graphite can be explained by a more thermally stable structure (TGA analysis) and increased density, which limited the phenomenon of foam melting before ignition. As a result, the distance between the cone and the sample was reduced to a lesser extent compared to the unmodified foam. Additionally, important is the structure of the foam, which was more porous for foams with graphite (SEM analysis), which facilitated the migration of heat into the material and changed the burning behavior. Analyzing the HRR charts, it was also observed that after the ignition of the VEF foam, there was a sharp increase in HRR, reaching a maximum of 123 kW/m^2^ after 54 s, and then a rapid decrease, creating a typical burning pattern of flexible foams with a single peak [19,20]. This course is similar to the heat rate versus time model during combustion of samples of polymeric material with average thermal transmittance and noncarbonizing [92]. Interestingly, a similar model is presented for foams containing graphite with the smallest grain size. This is also confirmed by a small residue after burning these materials, at the level of unmodified foam—approximately 8–8.5%. Moreover, in this group of foams, no significant changes in the THR and EHC values were observed. The exception is the 6 php S graphite foam as the THR and EHC were reduced by 25% and 20.7%, indicating that this reduction in gas-phase combustion affected the THR reduction. EHC refers to the gas-phase burnout of the volatile gases released from the polymer during combustion, while PML refers to the carbonization effect in the condensed phase [93]. These effects can be quantified by reducing EHC and PML, respectively. The increased char yield decreased the release of flammable volatiles; in turn, a reduction of EHC indicates flame inhibition or fuel dilution. Therefore, for the reference sample EHC and THR of VEFS6, it was reduced to 79.3% and 75%, respectively, while the carbon residue slightly increased from 8 to 9%, which is a reduction of the released fuel to 98.9%. A slightly higher THR reduction to 75% compared to the calculated value (79.3% × 98.9% = 78.4%) indicates the presence of an additional protective effect of the char layer containing graphite. However, the calculated relative protection effect is small and amounts to 4.4% (1–95.6%) (79.3% × 98.9% × 95.6% = 75%) which compared to the charring effect (1.1%), and the flame inhibition effect (20.7%) indicates that that operation in the gas phase results in a reduction of THR. The 25.8% reduction in pHRR of the sample containing 6 php of S graphite is also attributed to the gas phase operation as the additional protective barrier effect was only 5.4%.

Similar relationships were observed in the case of foam containing 5 php of graphite with an average grain size. The PML and EHC of VEFL5 were reduced by 1.8% and 17.2%, respectively. This indicates that the reduction in THR to 68.8% is related to gas-phase activity (17.2%) and barrier effect (15.4%) (82.8% × 98.2% × 84.6% = 68.8%). In the case of the foam containing 5 php of graphite with the largest particle size, no additional protective effect was observed, which corresponds to the scale efficiency in the TGA analysis. As a result, this resulted in less THR reduction. While the addition of 5 php of graphite L and X resulted in a slight change in the combustion process and a reduction in pHRR by 27.6% and 35.9%, respectively, compared to the reference sample, increasing the content of L and X graphite to 10 and 15 php resulted in a significant change in the nature of combustion. In Figure 10b,c, it can be seen that the HRR peak for foams with 10 php of graphite L and X becomes flat and elongated in time, and their behavior during the test can be attributed to the model between intermediate thick noncharring material and thermally thick charring materials [92]. This is confirmed by a significant reduction in the pHRR of the VEFL10 and VEFX10 foams, by 48.3% and 61.2%, respectively, compared to VEF, and the recorded times of achieving the maximum HRR after ignition, equal to 79 and 99 s, respectively. The greater reduction in pHRR of the foam containing graphite X is attributed to the greater ability of this graphite to expand and create an insulating layer on the surface of the foam, providing a strong protective barrier [34]. Comparing the fuel reduction (10.3%) and the reduction in the EHC of the combustible volatiles (4.6%), the calculated relative protection effect of 54.6% confirms that the formation of a strong protective barrier graphite is the main mechanism in reducing the pHRR of VEFX10 foam.

On the other hand, the addition of 15 php of graphite causes another change in the behavior of the foams during smoking. It is visible in the recorded HRR curves as a function of time in the form of flat HRR peaks and significantly extended in time. In addition, a characteristic pattern of the carbonizing and debris-forming foam model can be observed, with the first initial peak before charring and the second at the end of the measurement related to sinter cracking and pyrolysis [92]. Again, the lower value of pHRR = 18.5 kW/m^2^, which was achieved within 109 s from ignition, was characteristic for the sample containing X graphite with the largest grain size and the highest expansion coefficient, which was the largest reduction of pHRR, by 84.9% compared to VEF, among foams containing only graphite. In addition, the other data summarized in Table 9 regarding the VEFX15 foam confirms the best flame resistance of this foam, which is in line with the LOI values and UL94 tests. Compared to the unmodified EHC foam, it has been reduced to 65.5%. The increased residue (27.8% instead of 8.0%) reduced the amount of flammable gases to 78.5%. The THR value of the VEFX15 sample was reduced to 53.1%, which is consistent with the calculated value (65.5% × 78.5% = 51.4%). This indicates that the main mechanism of THR reduction is flame inhibition (34.5%) and the charring effect in the condensed phase (21.5%). In turn, the pHRR was reduced to 15.1%. The additional reduction in pHRR can be caused by the protection effect of the residues comprising graphite (TGA analysis), which produces an intumescent and dense char that acts as a physical barrier. The relative protection effect was calculated to be 70.7% (65.5% × 78.5% × 29.3% = 15.1%). The obtained results indicate that mainly barrier effect decreased the pHRR value.

For the sample containing 30 php BCP, there was a slight increase in pHRR to 124 kW/m^2^. Moreover, this material was characterized by an increased value of THR and EHC, by 51.6% and 26%, respectively, which is typical for foams containing natural fillers and with higher density [54]. Moreover, this sample burned more rapidly than the VEF foam as the peak HRR was reached in a shorter time, which confirms the increased flammability of this foam and is in line with the LOI and UL94 results. The organic nature of the filler made this sample behave like a polymeric material with medium thermal transmittance and noncarbonizing. On the other hand, the additional introduction of graphite resulted in a decrease in most of the parameters compared to the reference sample. The samples containing graphite and BCP were characterized by a low ignition time in the range of 7–9 s, depending on the type of graphite. In the case of the VEFBCP30S6 sample, the course of HRR changes as a function of time is characteristic between intermediate thick noncharring material and thermally thick charring materials [93]. While the samples containing 30 php BCP and 15 php of L and X graphite showed the course of HRR changes as a function of time typical for charring materials. Among these materials, better results were obtained for the foam containing 30 php BCP and 15 php graphite X, which is in line with the TGA, LOI, and UL94 analysis and confirms the highest flame resistance of this foam among the samples produced. Moreover, it was found that with this foam, an additive effect was obtained between BCP and graphite in reducing all parameters. For example, the pHRR compared to VEF, VEFX15, and VEFBCP30 decreased by 87.4%, 16.2%, and 87.5%, respectively. PML and EHC decreased by 31.1% and 42.5% compared to the reference sample. This corresponds to a reduction of the amount of released fuel to 68.9% and a reduction of the combustible volatiles to 67.5%. In turn, the pHRR was reduced to 12.6% compared to the VEF sample, indicating that the strong barrier effect (65.4%) (68.9% × 67.5% × 34.6% = 12.6%) is the primary flame-retardant mechanism for this sample.

#### 3.7.3. Fire Growth Rate Index

To assess the prediction of the rate of fire spread, a fire growth rate index is also currently used, which is determined from cone calorimetry data. The fire growth rate index (FIGRA) is defined as the ratio of the peak HRR to the time it is achieved. It allows one to estimate the size of a possible occurrence of a fire. A smaller FIGRA indicates the slower flame spread, which in effect means that there is more time to evacuate and provide the necessary fire extinguishing equipment. The calculated FIGRA values confirm the conclusions about the effectiveness of the different graphite types. Composites containing 15 php of graphite X and L, as well as composites that additionally contain BCP, show the lowest fire growth rate index. This is due to a significant decrease in pHRR and an increase in the time to reach its maximum from 90 s for VEF to 115 and 125 s for VEFL15 and VEFX15, respectively. It is interesting to note that composites containing from 3 to 5 php graphite with the smallest grain size, whose pHRR is smaller than the reference sample, show a fire growth rate index higher than the VEF sample. However, such FIGRA values are in accordance with previous conclusions. Composites containing graphite S are characterized by a percentage mass loss at the level of the reference sample (MLC analysis) and a small residue after thermolysis (TGA analysis), which indicates a weak effect in inhibiting flammability in the condensed phase, in contrast to samples containing graphite with a larger grain size. As a result, the time to reach the pHRR is 30 s shorter than the VEF samples, which resulted in an increase in FIGRA.

## 4. Conclusions

In this work, we analyzed the effects of EG with different particle size and expansion ratio and blackcurrant pomace on the viscoelastic properties of polyurethane foams. On the basis of the obtained results, we found that the amount and type of fillers affected the phase structure of the polyurethane matrix. Water introduced with fillers changes the thermal degradation behavior of composites compared to viscoelastic foam. The effect of filler particle size becomes clear only when more than 6 php EG was introduced. The graphite with a higher expansion ratio significantly increases the thermal stability of the composites. In contrast, the thermal stability of foams containing BCP and BCP with graphite significantly decreased in comparison to unmodified foam. Despite this, the char yield of foams containing BCP and BCP and graphite was higher than that of unmodified foam, which may indicate that BCP promotes the formation of a thermally stable coke inhibiting heat and mass transfer. Such a mechanism has provided a significant improvement in the flame resistance of viscoelastic polyurethane foam composites. While the addition of 30 php BCP in the foam resulted in even a slight deterioration of its flame resistance, the use of BCP together with graphite X led to the highest increase in LOI from 18.0 to 26.0% and the highest reduction in pHRR from 122.8 to 15.5 kW/m^2^. This effect of both fillers on the flammability of PUR composites is due to the formation of a char layer on the foam surface, which forms a strong thermal insulation and oxygen barrier, as evidenced by a significant reduction in PML and EHC, by 31.1% and 42.5%, respectively, compared to the reference sample. Analysis of SEM images indicates that the addition of BCP and BCP together with graphite causes changes in the pore structure of foams compared to unfilled foam and foams containing only graphite. It was observed that a significant BCP content causes the formation of pores of irregular shape and size and the formation of cracks in some struts and cell wall collapse. This can be explained by the fact that, together with BCP, significant amounts of OH groups present on the surface and inside the filler are introduced, which disturbs the reaction between functional groups and results in sites in the mixture with differential growth of foam cells. Changing the structure of the foams leads to a change in the density and mechanical properties of the foams determined in compression. As the graphite content increases, the density increases. Moreover, the addition of BCP causes a significant increase in density, which is due to the significant density of the fillers. However, the composites containing BCPs and graphite have a lower density than that of the fillers. This result is consistent with the SEM analysis, which showed the formation of larger pores for composites containing fillers. Despite this, the addition of BCP together with graphite L and X resulted in the greatest increase in hardness of the foams by 10% and 16%, respectively, compared to the reference sample. Advantageously, the permanent deformation of these composites was also the lowest. The obtained results of the flame resistance and mechanical properties of viscoelastic polyurethane foams containing BCP and expanded graphite indicate that blackcurrant pomace, which can be used as low-cost and biodegradable natural fillers when added to EG, improves the functional properties of VEF through additive effect.

## Figures and Tables

**Table 1:** The materials used to prepare the viscoelastic polyurethane foams (VEF).

Trade Name	Component	Manufacturer
Daltocel F442	polyetherol with functionality 3, hydroxyl number 42 mg KOH/g, ethoxy (EO) group content approximately 76%	Huntsman, TX, USA
Daltocel F526	polyetherol with functionality 3, hydroxyl number 126 mg KOH/g, molecular weight 1300 g/mol, EO group content over 70%	Huntsman, TX, USA
Rokopol M1170	polyetherol with functionality 3, hydroxyl number 35 mg KOH/g, EO group content over 50%	PCC Rokita SA, Brzeg Dolny, Poland
Rokopol F3600	polyetherol with functionality 3, hydroxyl number 48 mg KOH/g, molecular weight 3600 g/mol	PCC Rokita SA, Brzeg Dolny, Poland
Ongronat 4040	Isocyanate: a commercial mixture of mixture 4,4′-diphenylmethane diisocyanate and o-(p-isocyanarobenzyl) phenylisocyanate and polyisocyanate polyphenylmethane, functionality 2, NCO groups content 32.4%	BorsodChem, Kazincbarcika, Hungary
Jeffcat DPA	gelation catalyst: (*N*-(3-dimethylaminopropyl)—*N*,*N*-diisopropanolamine)	Huntsman, TX, USA
Jeffcat ZF10	foaming catalyst: (*N*,*N*,*N*′-trimethylethyl-*N*′-hydroxyethyl-bisaminoethyl ether)	Huntsman, TX, USA
Tegostab B4900	surfactant: polyether modified polysiloxane,	Evonik Industries AG, Essen, Germany

**Table 2:** Characteristics of expanded graphite (EG).

Symbol	Type	Minimum Carbon Content, [%]	Particle Size, [mm]	Expansion, [mL/g]	Maximum Volatile Matter Content, [%]	Bulk Density, [g/cm^3^]
EG S	EG 096	96	0–0.15	40–80	<10	~0.62
EG L	EG 290	90	0.2–0.6	200–300	<15	~0.64
EG X	EG 399	99	0.4–1.0	250–400	<15	~0.70

**Table 3:** Composites of viscoelastic polyurethane foams with additives.

Sample Symbol	Content in Polyol, php of Polyol				Content in Foam, wt. %				
	EG			BCP	EG			BCP	Matrix
	S	L	X		S	L	X		
VEF	0	0	0	0	0	0	0	0	100.00
VEFS3	3	0	0	0	1.9	0	0	0	98.1
VEFS4	4	0	0	0	2.5	0	0	0	97.5
VEFS5	5	0	0	0	3.1	0	0	0	96.9
VEFS6	6	0	0	0	3.7	0	0	0	96.3
VEFL5	0	5	0	0	0	3.1	0	0	96.9
VEFL10	0	10	0	0	0	6.0	0	0	94.0
VEFL15	0	15	0	0	0	8.7	0	0	91.3
VEFX5	0	0	5	0	0	0	3.1	0	96.9
VEFX10	0	0	10	0	0	0	6.0	0	94.0
VEFX15	0	0	15	0	0	0	8.7	0	91.3
VEFBCP30	0	0	0	30	0	0	0	16.0	84.0
VEFBCP30S6	6	0	0	30	3.1	0	0	15.5	81.4
VEFBCP30L15	0	15	0	30	0	7.3	0	14.8	77.9
VEFBCP30X15	0	0	15	30	0	0	7.3	14.8	77.9

**Table 4:** The results of the analysis of Fourier transform infrared spectroscopy (FTIR) spectra fragments in the range of bands of carbonyl groups.

Sample Symbol	R	DPS	UA, [%]	UR, [%]
VEF	1.02	0.50	38.2	61.8
VEFS6	1.44	0.59	42.8	57.2
VEFL15	1.24	0.55	45.2	54.8
VEFX15	1.03	0.51	45.6	54.4
VEFBCP30	1.22	0.54	44.6	55.4
VEFBCP30S6	1.58	0.61	48.4	51.6
VEFBCP30L15	1.81	0.64	48.9	51.1
VEFBCP30X15	1.99	0.67	49.1	50.9

**Table 5:** Results of the thermogravimetric analysis under nitrogen for the VEF composites.

Sample Symbol	m_150_, [%]	T_5%_, [°C]	Δm_1_, [%]	T_max1_, [°C]	V_max1_, [%/°C]	Δm_2_, [%]	T_max2_, [°C]	V_max2_, [%/°C]	Δm_3_, [%]	T_max3_, [°C]	V_max3_, [%/°C]	m_600_, [%]
VEF	0.3	278	14.1	304	0.30	18.6	338	0.43	57.0	413	1.23	10.0
VEFS3	0.3	284	15.5	311	0.29	17.4	345	0.37	55.6	418	1.26	11.2
VEFS4	0.3	280	14.7	309	0.30	18.2	343	0.38	55.1	419	1.27	11.5
VEFS5	0.3	280	13.2	305	0.26	19.8	344	0.39	54.9	419	1.24	11.7
VEFS6	0.3	284	12.9	307	0.27	20.8	345	0.42	54.1	420	1.24	11.9
VEFL5	0.3	282	11.2	310	0.25	21.8	348	0.37	55.3	420	1.25	11.7
VEFL10	0.3	286	11.5	311	0.26	21.3	342	0.40	54.3	420	1.25	12.2
VEFL15	0.3	284	11.9	*	*	19.8	339	0.48	55.1	418	1.17	12.8
VEFX5	0.3	287	12.9	310	0.30	20.7	343	0.41	54.7	416	1.26	11.4
VEFX10	0.3	284	13.7	*	*	20.7	339	0.48	51.8	416	1.18	13.5
VEFX15	0.3	282	14.1	*	*	20.5	338	0.48	49.0	415	1.11	15.8
BCP	5.8	127	21.7	289	0.34	16.1	334	0.38	31.8	407	0.36	24.5
VEFBCP30	1.6	252	14.3	302	0.28	20.3	340	0.40	45.2	418	1.01	12.7
VEFBCP30S6	1.7	259	13.8	*	*	20.1	342	0.38	46.3	420	0.97	14.2
VEFBCP30L15	1.7	249	13.5	*	*	20.6	331	0.41	45.5	416	0.96	15.6
VEFBCP30X15	1.4	253	13.3	*	*	21.2	335	0.42	43.2	417	0.91	17.9

* The maximum peak is impossible to determine; Δm_1_ = (m_1_ − m_150_), Δm_2_ = (m_2_ − m_1_), and Δm_3_ = (m_600_ − m_2_).

**Table 6:** Results of DSC thermogram analysis of the tested foams.

Sample Symbol	T_g1_ [°C]	T_g2_ [°C]	T_d1_ [°C]	ΔH_d1_ [J/g]	T_g1′_ [°C]	T_g2′_ [°C]	T_d1′_ [°C]	ΔH_d1′_ [J/g]
VEF	−63.8	-	68.5	56.9	−62.9	-	57.9	33.7
VEFS3	−62.9	-	54.6	52.7	−63.2	-	61.2	40.7
VEFS4	−63.1	-	50.4	46.8	−62.5	-	44.5	35.1
VEFS5	−62.5	-	60.1	46.7	−62.6	-	43.4	33.2
VEFS6	−64.2	-	65.6	46.2	−62.7	-	42.4	31.9
VEFL5	−63.1	−6.0	46.3	39.6	−63.1	−5.9	43.8	30.2
VEFL10	−62.8	−5.7	45.7	48.5	−63.4	−6.0	45.2	36.4
VEFL15	−62.8	−5.6	53.8	55.4	−63.3	−6.0	52.3	43.9
VEFX5	−63.7	−3.6	52.1	42.1	−63.5	−3.8	46.2	30.3
VEFX10	−62.3	−3.5	52.0	41.1	−62.4	−4.7	45.9	30.2
VEF X15	−62.6	−3.1	45.6	46.8	−63.4	−3.2	45.2	35.0
VEFBCP30	−65.5	-	100.1	60.4	−64.6	-	63.2	28.6
VEFBCP30S6	−59.9	-	60.2	34.5	−59.7	-	54.6	25.0
VEFBCP30L15	−59.6	-	75.3	75.4	−59.0	-	70.4	70.5
VEFBCP30X15	−60.3	-	85.6	86.0	−59.5	-	78.5	80.3

**Table 7:** Results of the analysis of apparent density and properties of foams determined during compression.

Sample Symbol	D, [kg/m^3^]	H, [kPa]	Cf	ε_50,_ [%]	ε_75,_ [%]	ε_90,_ [%]
VEF	39.7 ± 0.5	2.28 ± 0.11	2.09 ± 0.06	2.1 ± 0.2	10.0 ± 3.5	85.2 ± 12.8
VEFS3	42.7 ± 0.6	2.34 ± 0.18	2.24 ± 0.08	2.3 ± 0.4	12.5 ± 3.9	17.9 ± 3.6
VEFS4	43.3 ± 0.5	2.33 ± 0.11	1.98 ± 0.06	1.8 ± 0.5	2.5 ± 0.9	12.4 ± 0.3
VEFS5	43.7 ± 0.6	2.24 ± 0.10	2.23 ± 0.09	1.5 ± 0.3	3.1 ± 0.8	10.3 ± 0.6
VEFS6	43.9 ± 0.4	2.42 ± 0.13	2.11 ± 0.08	0.8 ± 0.1	1.3 ± 0.3	3.7 ± 0.4
VEFL5	44.4 ± 0.7	2.18 ± 0.08	2.26 ± 0.12	2.2 ± 0.1	2.4 ± 0.4	74.2 ± 5.6
VEFL10	47.1 ± 0.9	2.32 ± 0.13	2.23 ± 0.12	2.8 ± 0.1	10.3 ± 1.1	26.2 ± 4.5
VEFL15	48.9 ± 1.5	2.43 ± 0.12	2.24 ± 0.09	2.9 ± 0.2	10.5 ± 1.5	8.0 ± 0.9
VEFX5	42.4 ± 0.9	2.19 ± 0.14	2.27 ± 0.08	5.9 ± 0.6	5.9 ± 0.9	8.9 ± 0.5
VEFX10	43.9 ± 0.8	2.29 ± 0.13	2.29 ± 0.09	4.9 ± 0.5	9.9 ± 1.5	86.7 ± 9.8
VEFX15	47.9 ± 0.6	2.46 ± 0.14	2.33 ± 0.07	4.2 ± 0.7	5.4 ± 0.6	68.6 ± 7.5
VEFBCP30	52.2 ± 1.5	2.30 ± 0.18	2.60 ± 0.08	3.9 ± 0.5	6.2 ± 0.9	62.0 ± 7.2
VEFBCP30S6	54.8 ± 1.4	2.10 ± 0.19	2.66 ± 0.09	1.8 ± 0.5	2.5 ± 0.9	82.4 ± 0.3
VEFBCP30L15	57.5 ± 1.7	2.51 ± 0.17	2.59 ± 0.12	1.5 ± 0.3	3.1 ± 0.8	10.3 ± 0.6
VEFBCP30X15	57.1 ± 2.0	2.65 ± 0.19	2.65 ± 0.10	0.8 ± 0.1	1.3 ± 0.3	3.7 ± 0.4

D—apparent density, H—hardness, Cf—comfort factor, ε_50_—permanent deformation after compression by 50%, ε_75_—permanent deformation after compression by 75%, ε_90_—permanent deformation after compression by 90%, and ±—standard deviation.

**Table 8:** Limiting oxygen index (LOI) analysis and UL94 test results.

Sample	LOI, [%]	UL 94			
		Flammability Class	Loss of Weight [%]	Burning Time ^1^ [s]	Burning Speed [mm/min] ^2^
VEF	18.0	HB75	Burned	103	43.5
VEFS4	19.0	HB75	Burned	64	70.3
VEFL15	22.0	HB40b	18.3	10	-
VEFX15	23.6	HB40b	21.0	10	-
VEFBCP30	17.4	HB75	Burned	70	64.3
VEFBCP30L15	23.7	HB40b	23.7	15	-
VEFBCP30X15	26.0	HB40a	21.3	-	-

^1^ time recorded in the horizontal test only when the flame exceeds 25 mm from the edge of the sample on which the burner is placed; ^2^ was only calculated in the horizontal test if the flame exceeded the measuring distance between 25 and 100 mm of sample length.

**Table 9:** Summary of the results of the analysis using the mass loss calorimeter.

Sample Symbol	TTI [s]	THR [MJ/m^2^]	PML [%]	MLR [g/s]	HRR [kW/m^2^]	HRRm [kW/m^2^]	THRRm [s]	EHC [MJ/kg]	FIGRA [kW/m^2^s]
VEF	36	6.4	92.0	0.079	79.6	122.8	90	8.7	1.36
VEFS3	19	5.9	92.1	0.088	75.6	110.9	62	7.7	1.79
VEFS4	14	6.8	91.8	0.058	54.7	95.6	62	9.0	1.54
VEFS5	9	6.5	92.0	0.061	56.9	93.5	60	8.6	1.56
VEFS6	14	4.8	91.5	0.054	59.0	91.1	70	6.9	1.30
VEFL5	18	4.4	90.3	0.067	57.3	88.9	65	7.2	1.37
VEFL10	16	5.0	84.4	0.041	40.8	63.5	97	8.2	0.65
VEFL15	6	4.3	77.8	0.025	21.1	27.3	115	7.7	0.24
VEFX5	13	6.0	89.9	0.048	45.3	78.7	73	8.1	1.08
VEFX10	11	6.2	82.5	0.034	30.1	47.7	109	8.3	0.44
VEFX15	16	3.4	72.2	0.022	14.0	18.5	125	5.7	0.15
VEFBCP30	21	9.7	92.7	0.065	84.2	124.0	65	11.0	1.91
VEFBCP30S6	8	9.0	87.8	0.051	52.3	86.0	85	9.1	1.01
VEFBCP30L15	9	5.0	76.5	0.025	15.5	21.2	137	5.8	0.15
VEFBCP30X15	7	2.9	63.4	0.020	11.4	15.5	54	4.6	0.29

TTI—time to ignition; THR—total heat release; PML—percentage mass lost; MLR—mass loss rate; HRR—heat release rate; pHRR—peak heat release rate; TpHRR—time to reach peak heat release rate; EHC—Effective heat of combustion; FIGRA—fire growth rate index.

## Data Availability

Not applicable.

## Supplementary Materials

The following are available online at https://www.mdpi.com/article/10.3390/ma14071801/s1, Figure S1. TG curves for VEF foams and their composites containing: (**a**) different amounts of EG L, (**b**) BCP and a mixture of BCP and EG X.
