# Supplementary material for: Effects of Various Types of Expandable Graphite and Blackcurrant Pomace on the Properties of Viscoelastic Polyurethane Foams
Source: Materials (Basel). 2021 Apr 6;14(7):1801. doi: 10.3390/ma14071801 (PMC8038687; doi:10.3390/ma14071801)
Supplement: Supplementary file 1 [file materials-14-01801-s001.pdf]

Suplementary files:

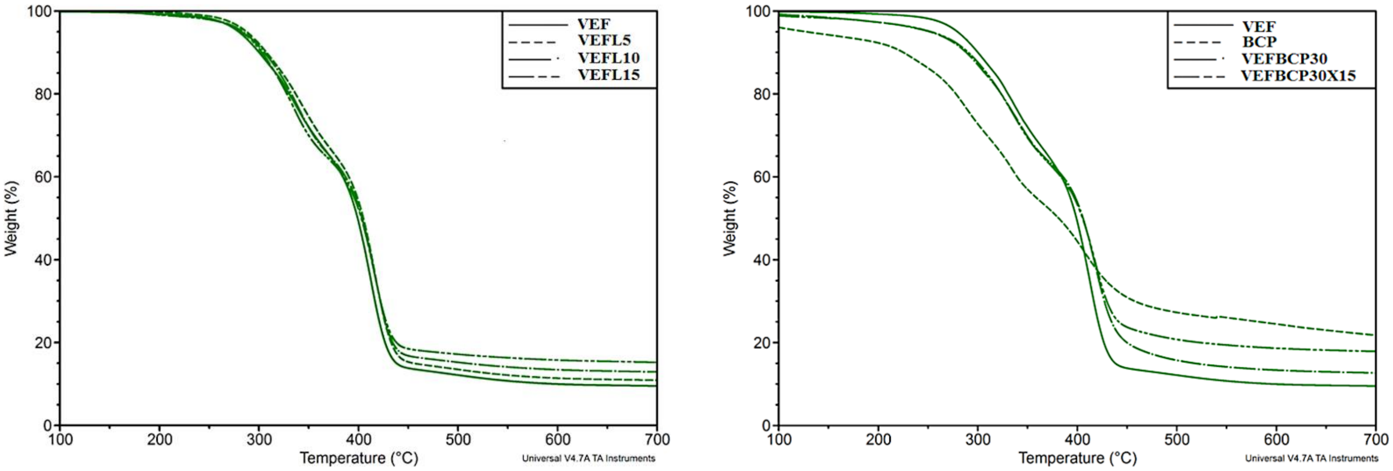

(a) (b)

Figure S1. TG curves for VEF foams and their composites containing: (**a**) different amounts of EG L, (**b**) BCP and a mixture of BCP and EG X.
